# Intra-articular Genicular Artery Pseudoaneurysm Mimicking Pigmented Villonodular Synovitis: A Case Report

**DOI:** 10.7759/cureus.113408

**Published:** 2026-07-26

**Authors:** Alaa Al-Taie, Rabia Bahauddin, Syed Alam, Renan Ibrahem Adam

**Affiliations:** 1 Radiology, Hamad General Hospital, Doha, QAT; 2 Radiology, Hamad Medical Corporation, Doha, QAT; 3 Medicine, Qatar University, Doha, QAT; 4 Musculoskeletal Radiology, Hamad Medical Corporation, Doha, QAT

**Keywords:** genicular artery, knee joint effusion, pigmented villonodular synovitis, pseudoaneurysm rupture, radiologic diagnostic pitfall

## Abstract

Pigmented villonodular synovitis (PVNS) is a non-aggressive entity involving the synovium. Magnetic resonance imaging (MRI) is regarded as the preferred imaging modality. MRI can occasionally be deceptive, with many benign conditions having a propensity for repeated bleeding intimately resembling PVNS. We present a case of an intra-articular pseudoaneurysm of the knee joint resembling PVNS on MRI. This 80-year-old male had multiple comorbidities, including a ruptured abdominal aortic aneurysm presented with recurrent knee swelling. The MRI showed complex hemorrhagic knee joint effusion with an intra-articular arterial branch pseudoaneurysm. The diagnosis was verified by CT angiography.

## Introduction

Atraumatic knee joint haemorrhage is an uncommon condition [1]. Pigmented villonodular synovitis (PVNS) is a non-neoplastic condition defined by one of two forms: localized or generalized overgrowth of synovium in the tendon sheaths, bursae, or joints. Evidence shows that it usually impacts the large joints, for instance the knee and the hip joint. Patients usually present with discomfort and swelling along with restricted range of motion combined with or without trauma [2]. On MRI, the intra-articular spectrum of diffuse PVNS is noted as a soft-tissue lesion with typical hypointense signal on T1-weighted images (T1WI) and T2- weighted images (T2WI) with blooming on gradient echo (GRE) sequences resulting from hemosiderin accumulation. Extrinsic osseous erosions can be noted. Entities that may resemble PVNS on diagnostic imaging encompass hemarthrosis, synovial chondromatosis, hemophilic arthritis, synovial hemangioma, and amyloid arthropathy [3]. Pseudoaneurysms and vascular injuries involving genicular articular branches are uncommon lesions that can result in recurrent hemarthrosis resembling MRI findings of PVNS [4]. We present an uncommon, noteworthy case of intra-articular arterial branch pseudoaneurysm causing repeated bleeding in the knee joint.

## Case presentation

**Figure 1:**
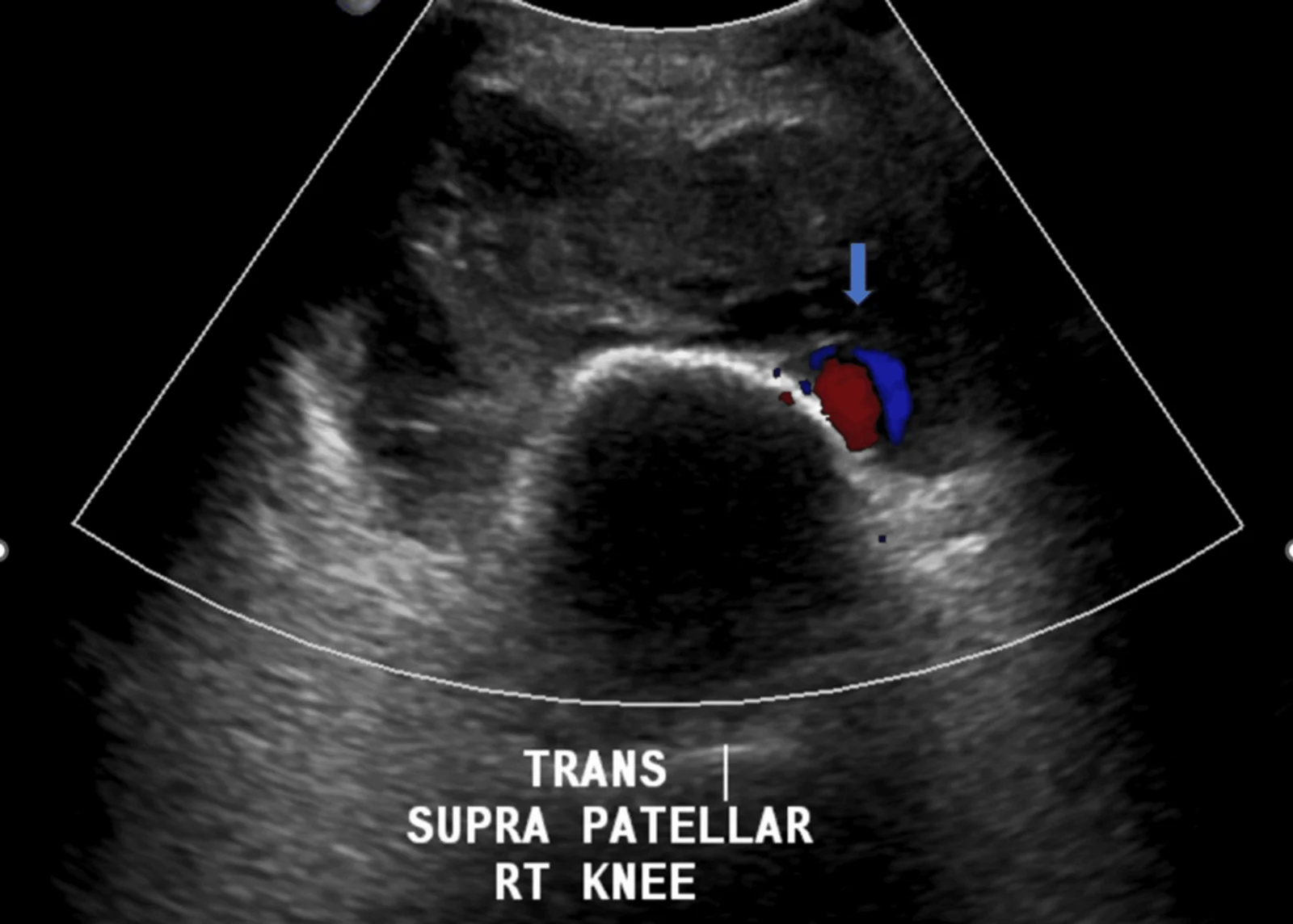
Ultrasound image of the knee Transverse ultrasound image of the knee demonstrating moderate iso- to hypoechoic complex suprapatellar joint effusion with synovitis. The blue arrow represents a focal yin-yang appearance on color Doppler images concerning for a small vascular lesion.

An 80-year-old male with a known condition of diabetes mellitus, hypertension, and coronary artery disease, including a ruptured abdominal aortic aneurysm, presented as an alert and oriented patient with right knee swelling. The swelling was progressively worsening with overlying skin color changes and mild tenderness noted. Ultrasonography (US) of the knee showed moderate joint effusion with internal septations. Findings were reported as a complex joint effusion with synovitis. Of note was a focal yin yang appearance concerning for a vascular lesion noted in the suprapatellar recess (Figure 1).

**Figure 2:**
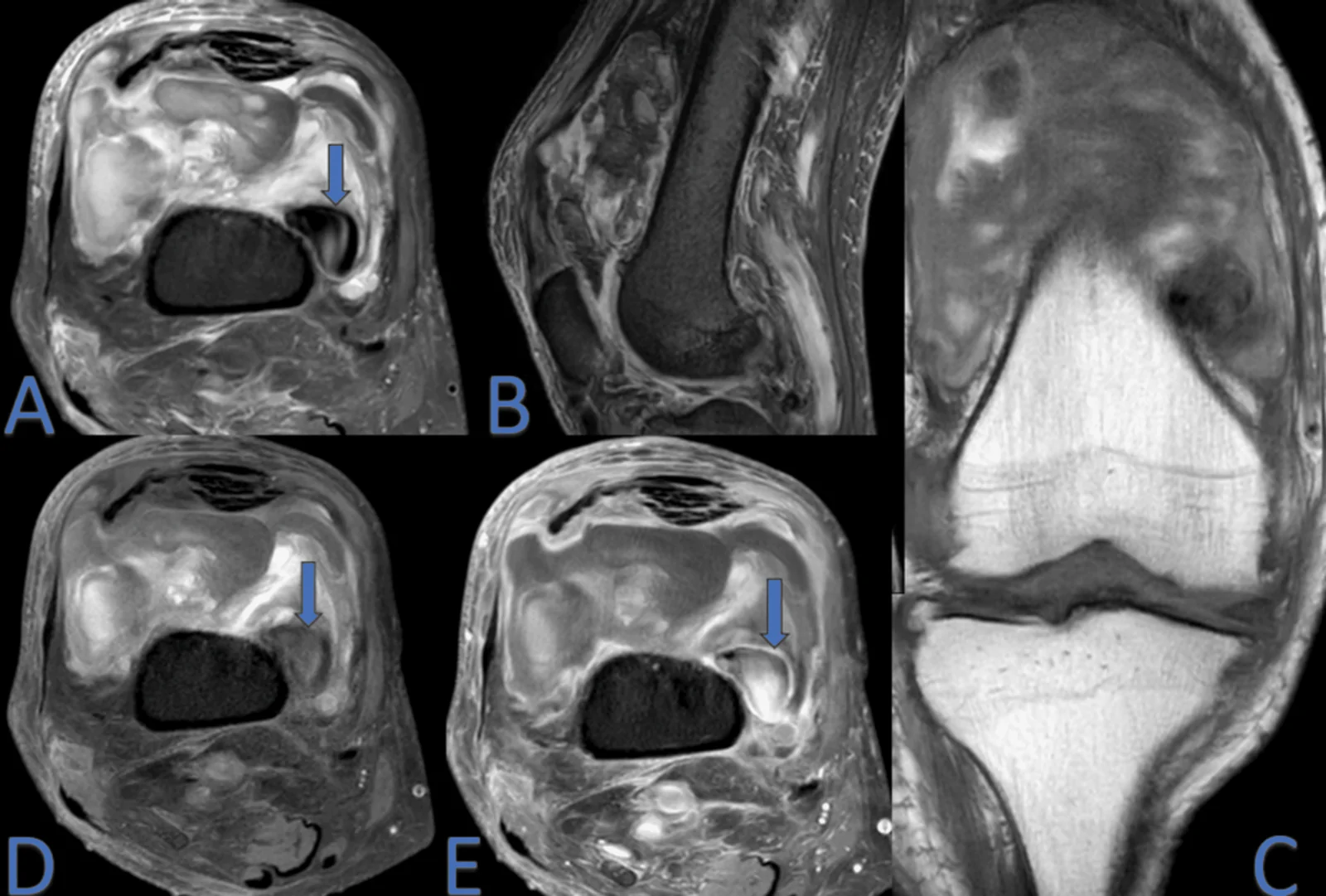
MRI images of the knee (A) Axial PD SPAIR, (B) Sagittal GRE, (C) Coronal T1, (D) Axial T1-weighted FS Pre-contrast, (E) Axial T1-weighted FS Post-contrast of the knee demonstrating large complex knee joint effusion, with high T1 signal and heterogeneous signal on fluid-sensitive sequences and extensive blooming artifact. Post-contrast heterogeneous enhancement is noted with synovitis. Arrow representing a small focal area of signal void with partial enhancement noted along the deep aspect of the suprapatellar region, demonstrating features consistent with pulsation artifact from an intra-articular vessel.

MRI was performed on the patient's knee, which redemonstrated the large complex knee joint effusion, predominantly in the suprapatellar recess. It showed heterogeneous hyperintense and predominantly hypointense signal on T1-weighted images (T1WI) and T2-weighted images (T2WI) with extensive blooming on gradient echo (GRE) sequences. Post-contrast synovial enhancement was noted with synovitis. Overall, the imaging findings were suggestive of PVNS; however, hemarthrosis was considered the more likely diagnosis. A small focal area of signal void on Proton Density with Spectral Attenuated Inversion Recovery (PD SPAIR)-weighted images with partial enhancement was noted in the suprapatellar region. It showed features of pulsation artifact, consistent with an intra-articular arterial branch aneurysm, likely from a branch of the medial genicular artery. No bony erosions were noted (Figure 2).

**Figure 3:**
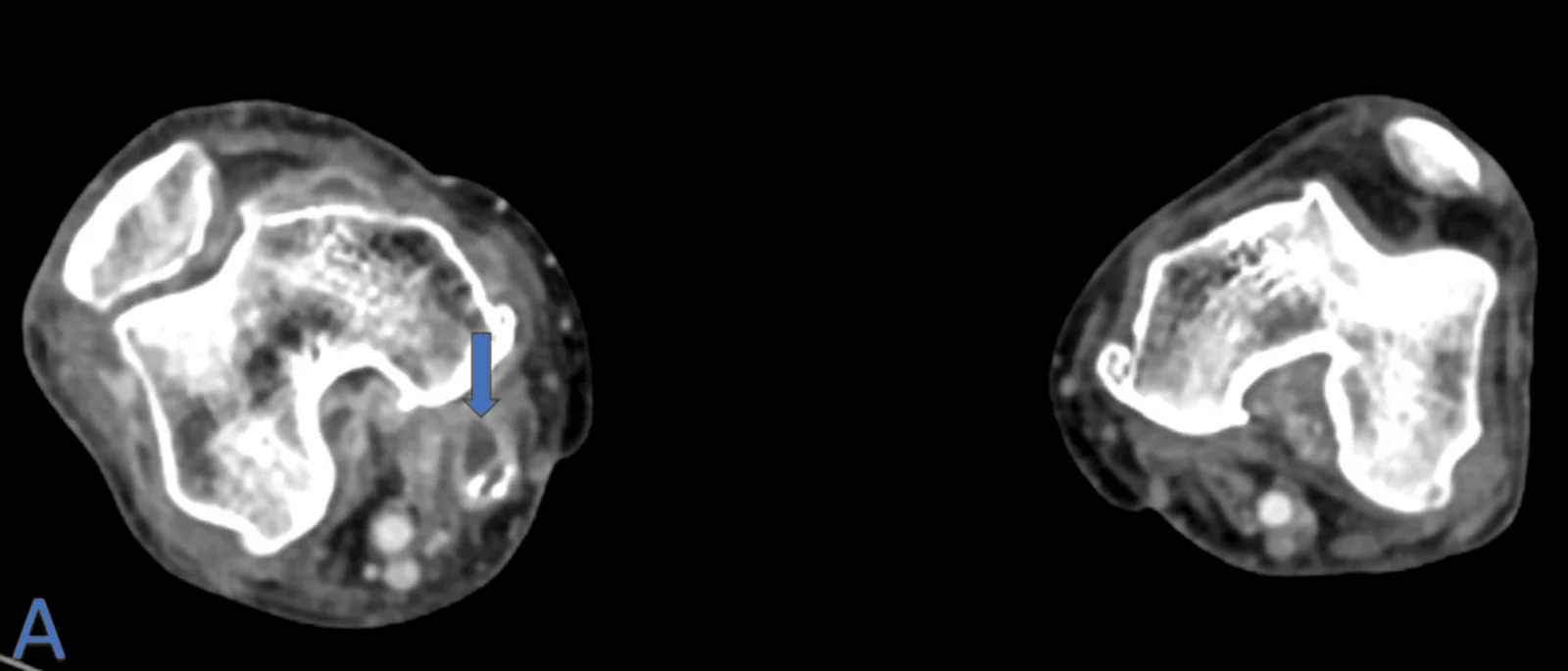
CT images of the knee (A) Axial CT of the knee with the arrow representing an oval contrast-filled structure that matches the contrast attenuation of the adjacent artery at the inferomedial joint space.

To confirm the pseudoaneurysm, CT angiography was performed. It showed a suprapatellar collection. An oval contrast-filled structure was seen at the superior joint space matching the contrast attenuation of the adjacent artery, suggestive of a pseudoaneurysm. No active contrast extravasation was noted (Figure 3). Hence, the pseudoaneurysm was verified.

**Figure 4:**
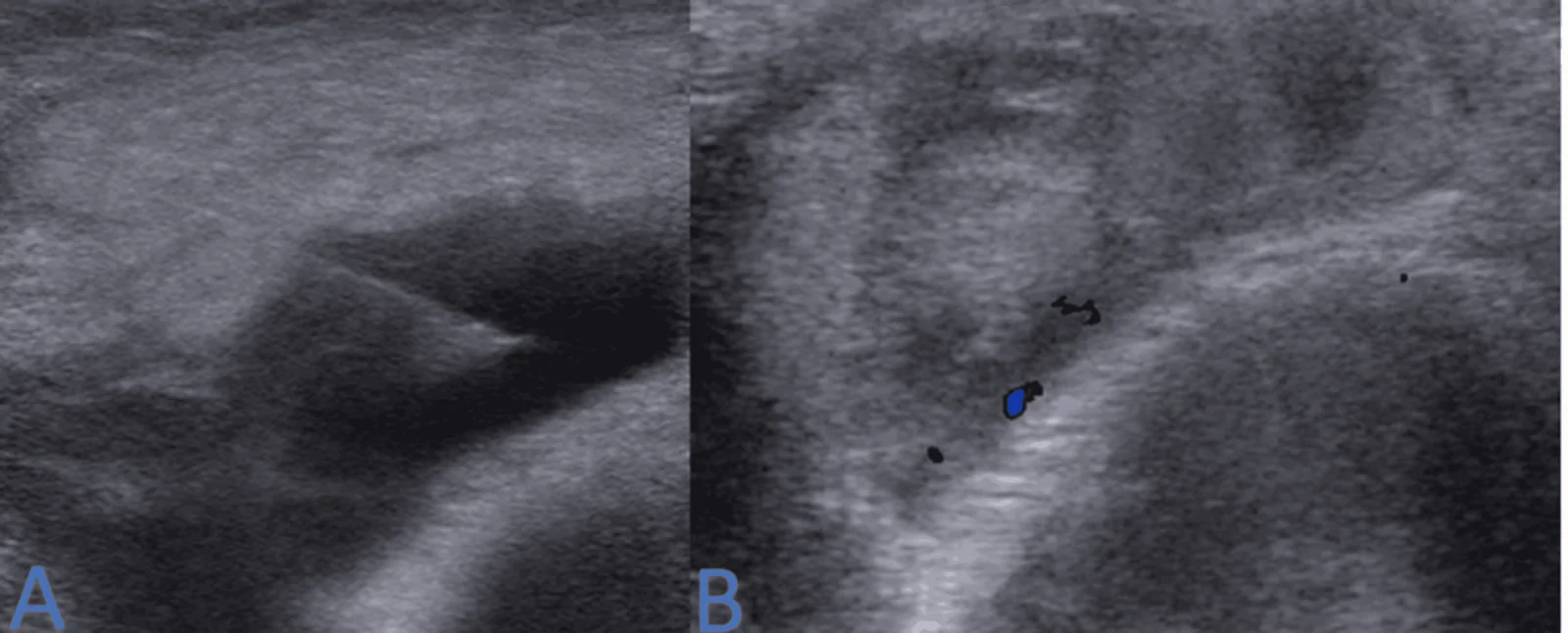
Ultrasound images of the knee (A), (B) Transverse ultrasound images showing a 22-gauge needle inserted into the genicular artery pseudoaneurysm with thrombin injection.

**Figure 5:**
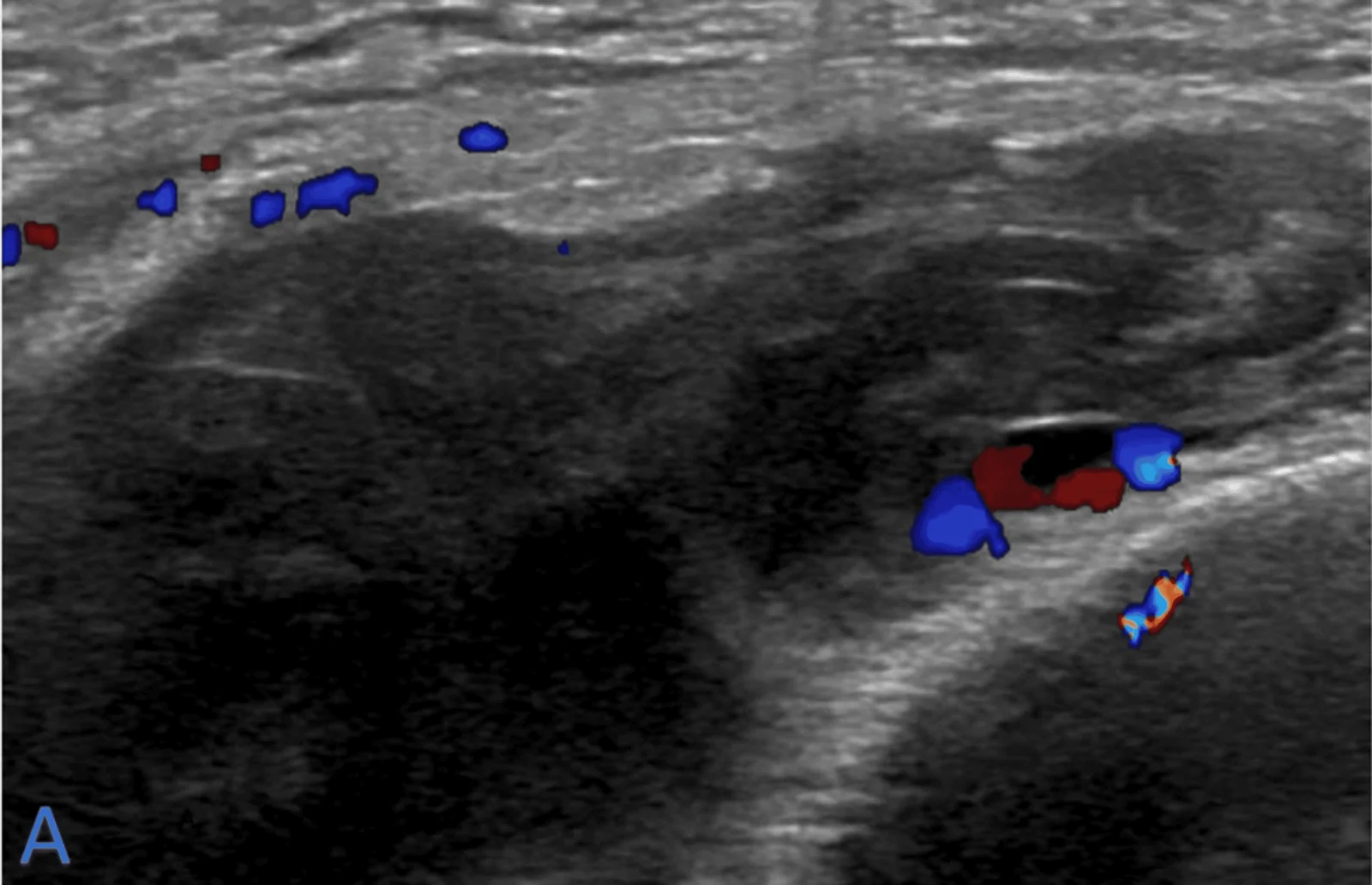
Follow-up ultrasound image of the knee Transverse follow-up ultrasound image showing a fairly heterogeneous subcutaneous collection at the medial aspect of the distal right thigh with a small linear vascular structure.

This was followed by thrombin injection by the interventional radiology team under ultrasound guidance (Figure 4). Doppler ultrasound was done the next day, which showed a thrombosed aneurysm with a small linear vascular structure likely signifying an adjacent artery (Figure 5). The patient was then mobilized and discharged with orthopedics follow-up. After six weeks, the patient was seen at the orthopedics clinic with decreased swelling, pain, and improved range of movement. The patient will continue follow-up in the orthopedic clinic.

## Discussion

The non-traumatic hemarthrosis of the knee in the advanced-age population is common in patients with osteoarthritis and was initially thought to be due to synovium [1]. PVNS is regarded as a neoplasm-related process. Several theories have been proposed regarding the pathogenesis of PVNS, including recurrent intra-articular hemorrhage and chronic inflammation; however, more recent evidence suggests that its independent growth potential and rare reports of malignant transformation support a neoplastic origin [3].

In our case, the patient had repeated knee swelling and underwent joint wash by orthopedics as a case of septic arthritis. Once the drain was removed, the swelling increased immediately, accompanied by noticeable changes in skin color. This was then followed by ultrasound, MRI, and CT angiography. The diffuse synovial thickening and hyperplastic changes in the synovium were secondary to the intra-articular hemorrhage from the pseudoaneurysm. The characteristic hypointense signal on T2-weighted images and blooming artifact on Gradient-Recalled Echo (GRE)-weighted images of the complex knee effusion closely mimicked soft tissue lesions of PVNS in our case. The foci of high T1 and T2 signal intensity seen in this complex knee joint effusion can also be seen in PVNS. They probably suggest the late subacute stage of blood breakdown products, likely favoring extracellular methemoglobin, with recent-onset bleeding [3].

All of these radiological findings closely resembled PVNS on MRI. The retrospective evaluation of the ultrasound along with the confirmation via CT-angiography guided us to the diagnosis of hemarthrosis due to pseudoaneurysm. Our case report emphasizes considering pseudoaneurysms as a differential diagnosis whenever there are features of PVNS on imaging, especially in older patients, those with trauma or operative procedures, and a good look at the history and old imaging. Cases of vascular trauma can also happen post-operative procedures, but are rare following knee arthroscopies [4]. Approximately 2% of pseudoaneurysm cases are attributed to musculoskeletal injury. In spite of the ease of treatment of pseudoaneurysms, they can cause notable bleeding if torn, requiring immediate intervention [4].

## Conclusions

A high level of concern should be present, especially in older age patients, for considering hemarthrosis due to pseudoaneurysms. Careful evaluation of the past medical and surgical history, along with any prior imaging, is critical as well. Although MRI is the preferred modality in most musculoskeletal conditions, the utilization of other imaging modalities like ultrasound, CT angiography, or Doppler in the suitable clinical context can avoid potential complications of biopsy.
